# Evaluation of different expression systems for the heterologous expression of pyranose 2-oxidase from *Trametes multicolor *in *E. coli*

**DOI:** 10.1186/1475-2859-9-14

**Published:** 2010-03-09

**Authors:** Oliver Spadiut, Gerald Posch, Roland Ludwig, Dietmar Haltrich, Clemens K Peterbauer

**Affiliations:** 1 Food Biotechnology Lab, Department of Food Sciences and Technology, BOKU - University of Natural Resources and Applied Life Sciences Vienna, Austria; 2 Research Centre Applied Biocatalysis, Graz, Austria; 3 School of Biotechnology, Royal Institute of Technology, Stockholm, Sweden; 4 Department of Nanobiotechnology, BOKU - University of Natural Resources and Applied Life Sciences Vienna, Austria

## Abstract

The heterologous production of the industrially relevant fungal enzyme pyranose 2-oxidase in the prokaryotic host *E. coli *was investigated using 3 different expression systems, i.e. the well-studied T7 RNA polymerase based pET21d^+^, the L-arabinose inducible pBAD and the pCOLD system. Preliminary experiments were done in shaking flasks at 25°C and optimized induction conditions to compare the productivity levels of the different expression systems. The pET21d^+ ^and the pCOLD system gave 29 U/L·h and 14 U/L·h of active pyranose 2-oxidase, respectively, whereas the pBAD system only produced 6 U/L·h. Process conditions for batch fermentations were optimized for the pET21d^+ ^and the pCOLD systems in order to reduce the formation of inactive inclusion bodies. The highest productivity rate with the pET21d^+ ^expression system in batch fermentations was determined at 25°C with 32 U/L·h. The pCOLD system showed the highest productivity rate (19 U/L·h) at 25°C and induction from the start of the cultivation. Using the pCOLD system in a fed batch fermentation at 25°C with a specific growth rate of μ = 0.15 h^-1^resulted in the highest productivity rate of active pyranose oxidase with 206 U/L·h.

## Background

Enzymatic catalysis provides tremendous opportunities for industry to carry out efficient and economical biocatalytic conversions. Traditional markets include the food and feed industry, but enzymatic processes are increasingly implemented in emerging markets such as fine chemical production and pharmaceutical industries [1]. Cost-efficient high-yield production of the biocatalysts, usually by heterologous expression in bacterial or yeast systems, is critical for the economic viability of such processes. The choice of the production host depends on several properties of the target protein itself. If posttranslational modifications are not required, prokaryotic systems like *E. coli *are most attractive due to high productivity, low cost and easy handling. However, overexpression of recombinant proteins in bacteria can cause problems like the production of insoluble aggregates of misfolded and inactive proteins called inclusion bodies (IB). Inclusion bodies consist of the overproduced polypeptide aggregated with small amounts of foreign proteins and nucleic acids [2]. Strategies for resolving this problem and for optimizing expression levels in *E. coli *have already been reported [3-7] and include the application of well-characterized expression systems under the control of tightly regulated promoters as well as the application of different expression conditions. In general, reduced cell growth, caused by lowered temperatures or oxygen limitation, promotes the expression of complex heterologous proteins in an active and soluble form and reduces IB formation [8-11]. Overdrive of inducer, on the other hand, results in IB formation and cell damage, whereas limited induction is favorable for native enzyme production [12]. Refolding of IB is widely discussed in literature [13,14], but is not possible for every given protein.

In this study we analyzed different expression systems for the production of recombinant pyranose 2-oxidase (POx; pyranose:oxygen 2-oxidoreductase; glucose 2-oxidase; EC 1.1.3.10) from *Trametes multicolor *in the prokaryotic host *E. coli*. POx is a homotetrameric flavoprotein, typically of a molecular mass of 270 kDa, with each of the four 68-kDa subunits carrying one covalently bound flavin adenine dinucleotide (FAD). It is widespread in wood-degrading basidiomycetes and catalyzes the oxidation of different aldopyranoses at C2 to the corresponding 2-ketoaldoses, producing H_2_O_2 _as a by-product [15-17]. In the past few years, POx has gained increasing attention for industrial applications, such as the synthesis of carbohydrate derivates and rare sugars [18], analytical applications [19], as well as for applications in enzymatic biofuel cells [20]. Cost-efficient enzyme supply is critical for any such application. The heterologous expression of POx in *E. coli *has been investigated before [21-23]. However, these studies only dealt with one expression system based on the vector pSE420 (Invitrogen) and improvement of POx expression by medium optimization and oxygen limitation, and enzyme yields remained limited due to IB formation.

It was the goal of this work to investigate different expression systems for POx in *E. coli *and to optimize the production in a bioreactor to minimize the formation of insoluble IB. Apart from the T7 RNA polymerase based pET21d^+ ^[24], we investigated the L-arabinose inducible pBAD [25,26] and the pCOLD expression system [27,28].

## Methods

### Microorganisms and cultivation

*Escherichia coli *BL21 DE3 and *E. coli *TOP10 cells were used for maintenance and propagation of plasmids and as hosts for expression studies. For propagation, *E. coli *cells were cultivated in TB_amp_-media (yeast extract 24 g/L; peptone from casein 12 g/L; glycerol 4 mL/L; KH_2_PO_4_-buffer 1 M, pH 7.5) under appropriate selective conditions (ampicillin was added to a final concentration of 0.1 g/L).

The inoculi for the different fermentations were cultivated in a defined Seed Stage Medium (glycerol 5 g/L; peptone from casein 5 g/L; yeast extract 5 g/L; NaCl 5 g/L; ampicillin 0.1 g/L). For cultivation and expression studies, *E. coli *cells were grown in a Production Stage Medium in the bioreactors (MPCGly-Medium: glycerol 10 g/L; peptone from casein 10 g/L; M9-Salts 5× conc. 200 mL/L). The M9-Salts 5× conc. solution (Na_2_HPO_4_·2H_2_O 42.5 g/L; KH_2_PO_4 _15.0 g/L; NaCl 2.5 g/L; NH_4_Cl 5.0 g/L) was prepared and autoclaved separately. After sterilization, the M9-Salts 5× conc. solution, 10 mL MgSO_4 _[1 M], 0.5 mL CaCl_2 _[1 M], 0.1 mL/L sterile antifoam and 0.1 g/L ampicillin were injected through a sterile filter into the bioreactor.

For fed-batch experiments a chemically defined Synthetic Medium was developed (glycerol 185.6 g/L; (NH_4_)_2_SO_4 _61.7 g/L; KH_2_PO_4 _5 g/L; Na_2_HPO_4_·2H_2_O 11.5 g/L; MgSO_4_·7H_2_O [1 M] 19 mL/L; CaCl_2_·6H_2_O [1 M] 10.5 mL/L; lactose monohydrate 5.25 g/L; trace element solution 10 mL/L; ampicillin 0.1 g/L). The trace element solution (FeCl_3 _anhydrous 4.00 g/L; MnCl_2_·4H_2_O 2.35 g/L; ZnSO_4_·7H_2_O 1.60 g/L; H_3_BO_3 _0.50 g/L; CoCl_2_·6H_2_O 0.40 g/L; (NH_4_)_6_Mo_7_O_24_·4H_2_O 0.40 g/L; CuCl_2 _anhydrous 0.25 g/L) was prepared separately, filter sterilized and added to the sterile medium. The chemicals used were of the purest grade available and purchased from Sigma (Vienna, Austria) and Roth (Karlsruhe, Germany).

Construction of the expression vectors pET21d^+^/POx, pBAD His A/POx and pCOLDIII/POx

Nucleotides, buffers and enzymes were purchased from Fermentas (St. Leon-Rot, Germany).

The construction of the pET21d^+^/POx vector (pHL2), which contains the His-tagged pyranose 2-oxidase gene from *Trametes multicolor *under the control of the T7 promoter, is described elsewhere [29].

To obtain a tightly regulated expression system we used the pBAD/His A vector (Invitrogen; Carlsbad, CA, U.S.A.) using the L-arabinose inducible araBAD promoter, which displays only very low transcription levels in the absence of arabinose. The POx-encoding gene from pHL2 was amplified using the primers BAD_*Pst*I_fwd (5'-aagaaggagctgcagcatggctacc-3') and BAD_*Eco*RI_rev (5'-gctcgagtggaattcctcactgagcc-3'). After double digestion with *Pst*I and *Eco*RI, the POx gene fragment was ligated into the *Pst*I/*Eco*RI digested pBAD His A vector at 16°C over night.

The cold-shock expression vector III (pCOLD III; Takara Bio Inc., Japan) contains the promoter of the cold-shock gene *cspA*. The POx-encoding gene from pHL2 was amplified using the primers COLD_fwd (5'-aggagatatcatatggctaccagctcg-3') and COLD_rev (5'-gttagcagcctgcagtcagtggtg-3'). Both the PCR fragment and the pCOLD III plasmid where double-digested with *Nde*I and *Pst*I and ligated at 16°C over night.

Correct insertion of the POx-encoding genes and the absence of mutations were checked by DNA sequencing, and verified plasmids were transformed into *E. coli *BL21 DE3 (pET21d^+^- and pCOLD-derivatives) and *E. coli *TOP10 (pBAD-derivative).

### Shake-flask cultivations

In order to compare the amount of soluble, active POx of the 3 different expression systems, preliminary experiments in baffled shake-flasks were carried out at 25°C. According to Kotik et al. [22] an induction with 0.5% lactose (w/v) for the pET21d^+ ^system was used. For the pBAD system the concentration of the inducer L-arabinose was varied from 0.001 to 1% (w/v) according to the manufacturer's recommendations. The comparative study at 25°C was then performed by inducing the cultures with an optimal concentration of 0.1% L-arabinose. Small-scale tests showed no difference in expression between induction with either IPTG (0.1, 0.5 and 1.0 mM) or lactose (0.5% w/v) using the pCOLD system, therefore the cheaper lactose was used for induction. All cultures were induced at an OD_600_~0.5 and cultivated at 25°C for 20 h. After centrifugation, cells were lysed using a French Press and POx activity was analyzed by the chromogenic ABTS assay [30].

### Preparation of the working cell bank and preculture

A single colony of transformed *E. coli *cells was used as inoculum for 50 mL TB_amp _in baffled flasks at 37°C and was grown to an optical density OD_600 _of ~1.0. To obtain the working cell bank, 500 μL of this cell suspension were mixed with 250 μL sterile 75% glycerol, frozen in liquid nitrogen and stored at -70°C.

As a preculture, Erlenmeyer flasks containing 250 mL sterile Seed Stage Medium were inoculated with 250 μL of working cell bench suspension and incubated at 37°C and 140 rpm. At an OD_600 _of ~0.5, this preculture (5% (v/v) of the total culture volume) was transferred into the bioreactor.

### Bioreactor studies

Batch cultivations were done in 42 L computer-controlled stirred tank reactors (Applikon, Schiedam, the Netherlands), with a working volume of 30 L. Culture pH was maintained at pH 7.0 by automatic addition of sterile NaOH [4 M] and the dissolved oxygen concentration (DO_2_) was set to 30%. The parameters were controlled using a Bio Controller ADI 1030 digital controller (Applikon) and online-monitored by a computer data logger (BioExpert NT, Applikon). DO_2 _levels were maintained by supplying filtered air automatically (0-15 L/min) and adjusting the stirrer velocity. Temperature, inducer concentration and time of induction were varied to optimize protein expression.

Fed-batch experiments were performed in a 7 L stirred tank reactor (MBR Bio Reactor AG, Wetzikon, Switzerland). Online process parameters were controlled by an IMCS-2000 digital control unit (PCS AG, Wetzikon, Switzerland). One hundred and twenty five mL of preculture were used to inoculate an initial batch phase volume of 2.5 L. Fed-batch experiments were operated at 25°C and the medium was always supplemented with lactose 0.5% (w/v) for induction of POx expression from the beginning of cultivation. Following glycerol depletion, which was indicated by a rapid increase of DO_2_, the fed-batch phase was started. Carbon-limited exponential feeding was carried out according to a predetermined model (formula for glycerol feed: y = 11.25 * e^0.15x^, R^2 ^= 1; x is the biomass concentration in g/L) using a P-1 peristaltic pump (Pharmacia Biotech, Uppsala, Sweden). The feed pump was operated manually and feed flow rates were adjusted every hour to maintain exponential feeding rates. High levels of glycerol had to be avoided in order to maintain a desired specific growth rate of μ = 0.15 h^-1^.

### Off-line analysis of parameters

Samples were taken in appropriate time intervals during fermentations and analyzed for optical density at 600 nm (OD_600_), dry cell weight (DCW) and concentration of the substrate glycerol and the inducer lactose. Likewise, POx activity and the total intracellular protein concentration were determined to evaluate the performance of the expression system. To evaluate the applied process conditions, the factor Yx/s was determined. Yx/s stands for the amount of biomass [g] which was produced per g of substrate.

Optical density was measured in duplicates. DCW [g/L] was determined by centrifuging 20 mL of cell suspension in a previously dried and pre-weighed centrifuge tube at 4000 rpm for 15 min. Lactose and glycerol concentrations in the supernatant were determined by using test kits (Hoffmann-La Roche Ltd, Basel, Switzerland; Megazyme International Ireland Ltd., Wicklow, Ireland) following the manufacturer's protocols, the cells were washed twice with 20 mL H_2_O dist. and DCW was determined after drying the wet pellet at 60°C to a constant weight.

POx activity was measured with the standard chromogenic ABTS [2,2'-azinobis(3-ethylbenzthiazolinesulfonic acid)] assay [30] from the sample crude extract after cell disruption using a French press. A sample of diluted enzyme (10 μl) was added to 980 μl assay buffer containing horseradish peroxidase (142 U), ABTS (14.7 mg) and KH_2_PO_4_-buffer (50 mM, pH 6.5). The reaction was started by addition of D-glucose (20 mM). The absorbance change at 420 nm was recorded at 30°C for 180 sec. The chromophore ε_420 _used was 42.3 mM^-1^·cm^-1^. One Unit of POx activity was defined as the amount of enzyme necessary for the oxidation of 2 μmol of ABTS per min (which equals the consumption of 1 μmol of O_2_) under the assay conditions. Kinetic constants were calculated by non-linear least-square regression, fitting the data to the Henri-Michaelis-Menten equation. Protein concentrations were determined at 595 nm by the Bradford assay [31] using the BioRad Protein Assay Kit (BioRad; Vienna, Austria) with bovine serum albumin (BSA) used in concentrations of 0.1 - 1.0 mg/mL for the standard calibration curve.

### Electrophoresis

After homogenization of the samples cell debris including IB was harvested by centrifugation (13 200 rpm, 4°C, 20 min). An aliquot of this pellet (100 mg) was washed 2 times with H_2_O dist., resuspended in H_2_O dist. and analyzed for the presence of IB by SDS-PAGE, as described by Laemmli [32]. To analyze the expression of soluble POx, 50 μl of crude extract were mixed with 50 μl Laemmli buffer, heated at 95°C for 5 min and analyzed. SDS-PAGE was performed using a 5% stacking gel and a 10% separating gel run in a PerfectBlue vertical electrophoresis system (Peqlab; Erlangen, Germany). Samples were diluted to ~1 mg of protein per millilitre and aliquots of 5 μl were loaded per lane. The molecular weight standard used was the Precision Plus Protein Dual Color (Biorad), and the gels were stained with Coomassie blue.

## Results and discussion

### Preliminary expression experiments at 25°C

**Figure 1:**
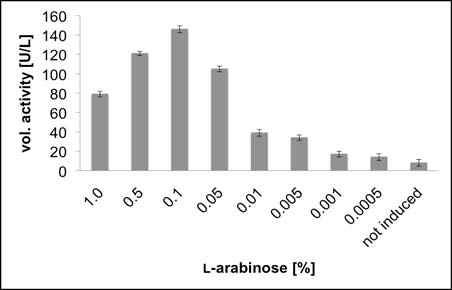
**Volumetric activity [U/L] of POx obtained in cultivations in baffled shaking flasks to analyze the optimal concentration of L-arabinose for induction of the pBAD HisA/POx expression system at 25°C**.

In order to compare the amount of soluble, active POx in the 3 different expression systems, preliminary experiments in baffled shake-flasks at 25°C were carried out. The pET21d^+ ^and the pCOLD system were induced by addition of 0.5% lactose (w/v); for the pBAD system, an L-arabinose concentration of 1 g/L (0.1% w/v, Fig. 1) resulted in the highest expression of active POx. Consequently, this concentration was used for further comparative studies.

**Figure 2:**
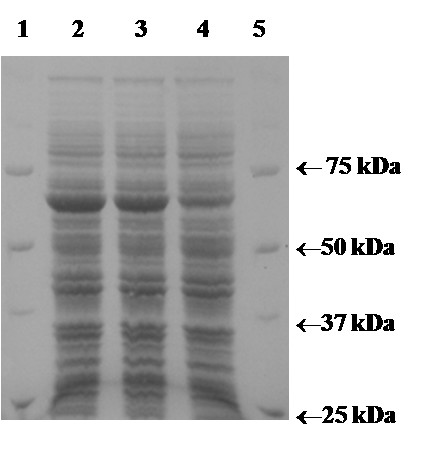
**SDS-PAGE analysis of crude extracts obtained in comparative expression studies at 25°C**. The three expression systems pET21d^+^, pBAD and pCOLD were optimally induced and cultivated for 20 h. One gram wet biomass of each cultivation was resuspended in 3 mL lysis buffer and lysed by the French Press. After centrifugation, 50 μl crude extracts were mixed with 50 μl Laemmli buffer, heated at 95°C for 5 min and 10 μl aliquots were analyzed by SDS-PAGE. Lane 1, molecular mass marker proteins; lane 2, pET21d^+^; lane 3, pCOLD; lane 4, pBAD; lane 5, molecular mass marker proteins.

In these experiments at 25°C and optimal induction, the pET21d^+ ^system showed the highest productivity rate of 29 U/L·h active, recombinant POx. With the pCOLD system, 14 Units active POx were produced per liter and hour. Possible effects of the temperature resulting in only half the yield compared to the pET21d^+ ^system were further investigated in batch fermentation studies. The tightly regulated L-arabinose inducible pBAD system showed a productivity rate of only 6 U/L·h, approximately 20% of the pET21d^+ ^system. Crude extracts, which were obtained by lysis of 1 g of wet biomass, were also analyzed for their content of expressed POx by SDS-PAGE (Fig. 2). The pBAD expression system was not competitive compared with pET21d^+ ^and pCOLD, and was not investigated further.

### Expression of POx using the pET21d^+ ^expression system

The expression vector pET21d^+ ^and other derivatives based on the T7 RNA polymerase promoter are widely used for the heterologous expression of proteins in bacteria. In previous reports, POx was shown to be highly susceptible to IB formation using the similar expression system pSE420 under the control of the *trc *promoter in *E. coli *[22,23]. Kotik et al. used 3 different temperatures (37°C, 25°C and 22°C) and oxygen limitation at 25°C to reduce IB formation, but the results were not satisfying. In the latter work, limited oxygen rates led to increased specific activity of POx, but the reduced growth rate μ_max _= 0.08 h^-1 ^resulted in very long cultivation times. Additionally, complete consumption of lactose may have resulted in a non-permanent induction of protein expression, which makes the data for the fed-batch experiments difficult to interpret.

Therefore, process conditions had to be optimized to minimize the formation of IB. Table 1 summarizes fermentation experiments to analyze the effect of temperature and time of induction on the expression of active POx. In all fermentations protein expression was induced by 0.5% lactose (w/v), which allowed continuous induction throughout the process.

**Table 1:** Important parameters and results of 6 different batch fermentations using the pET21d^+^/POx expression system.

fermentation	1	2	3	4	5	6
time of induction [h]	0	0	0	6 (OD_600 _= 0.7)	0	0
temp. [°C]	37	30	25	25	22	18
DCW [g/L]	4.5	4.6	4.6	3.2	5.7	4.1
Yx/s [g/g]	0.46	0.46	0.47	0.33	0.56	0.41
μ_max _[h^-1^]	1.15	0.88	0.48	1.25	0.23	0.20
vol. activity [U/L]	1	45	916	425	1155	1300
process time [h]	25	25	26	22	40	50
productivity [U/L·h]	0	2	32	18.5	28	26
space time yield [mg/L·h]	0	0.3	4.0	2.3	3.5	3.3

**Figure 3:**
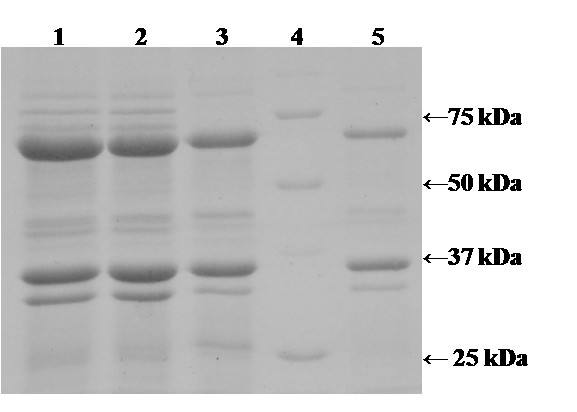
**SDS-PAGE analysis of IB formation during fermentations at different temperatures with the pET21d^+^/POx expression system**. After lysis, cell debris containing IB was collected by centrifugation; 100 mg cell debris were washed, resuspended in Laemmli Buffer, heated at 95°C for 5 min and 10 μl aliquots were loaded per lane. Lane 1, 37°C; lane 2, 30°C; lane 3, 25°C; lane 4, molecular mass marker proteins; lane 5, 20°C.

Fermentation 1 at 37°C was divided into a short lag phase and a very short exponential phase. Glycerol was depleted after 23 hours. Growth rate was very high (μ_max _= 1.15 h^-1^), but no active POx could be detected, only IB (Fig. 3). Fermentation 2 at 30°C was characterized by a short lag phase but a longer exponential phase compared to fermentation 1. Growth rate was still high (μ_max _= 0.88 h^-1^), but glycerol was not consumed quantitatively. IB formation was still high (Fig. 3), but active POx could be detected, demonstrating the pronounced effect of temperature on IB formation. Two further fermentations under these conditions were performed with lower inducer concentrations (0.05% and 0.005% lactose), but no effect on the expression level of active POx could be detected (data not shown).

**Figure 4:**
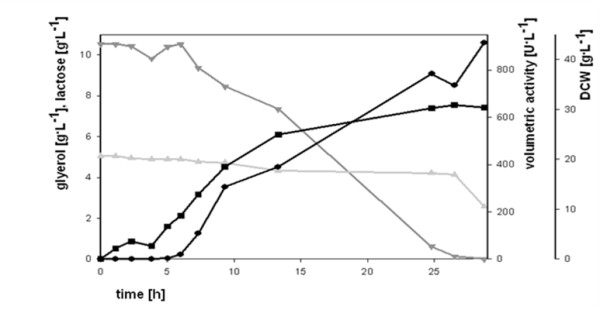
**Batch fermentation at 25°C and an induction with 0.5% lactose from the very beginning using the pET21d^+^/POx expression system**. (dark grey triangle), glycerol; (light grey triangle), lactose; (Black square), dry cell weight; (Black circle), volumetric activity of POx.

In order to further optimize the expression of active, recombinant POx in *E. coli*, additional fermentations at lower temperatures were performed with the same, defined inducer concentration of 0.5% lactose. Fermentations 3 and 4 were performed at 25°C with different induction times (Table 1). Glycerol depletion occurred after 26 and 22 hours, respectively. Induction at an OD_600 _of ~0.7 resulted in a much shorter exponential growth phase and a reduced productivity of less than half the amount of soluble, active POx. Production of active enzyme was apparently growth associated and directly proportional to the increase of OD_600 _(Fig. 4). Obviously, POx production did not cause a metabolic burden on *E. coli*, the achieved DCW and Yx/s were even higher in Fermentation 3 than in Fermentation 4. Therefore, as described previously also for other recombinant proteins [33], an early induction was possible and resulted in an increased amount of produced recombinant POx. Consequently, induction was started at the beginning of the cultivation in further experiments.

Fermentations 5 and 6 were done at 22°C and 18°C. The lower the temperature, the longer were the lag and the exponential growth phase. After 50 hours of process time still half of the substrate glycerol was left in fermentation 6, and the yield of soluble, active POx was increased up to 1300 U/L. Reduced temperature improved production of native enzyme and reduced IB formation significantly, but process times increased dramatically.

To determine an influence of carbon source limitation on the expression rate of active protein, a series of experiments with limited feed rates (linear and exponential) of glycerol were done. At 30°C a linear feed of 0.1 g glycerol/L·h resulted in a high growth rate of μ_max _= 0.80 h^-1 ^and formation of IB but no active POx. A glycerol feed, which increased according to the OD_600 _at 25°C, resulted in a growth rate of 0.47 h^-1^. Maximum volumetric activity was 834 U/L, due to the lower temperature (data not shown). The more complex fed-batch systems proved again the huge effect of the process temperature on the formation of active POx. By comparing μ_max _and the achieved volumetric activity between the batch and fed-batch systems (see above and Table 1), it became obvious that carbon source limitation was not a successful strategy to minimize the formation of IB.

**Figure 5:**
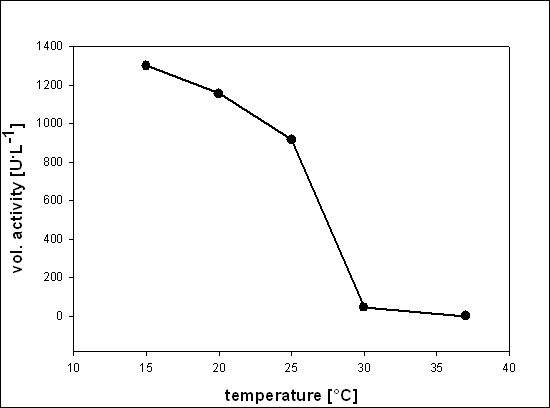
**Influence of the growth temperature during batch fermentations on the expression of active, recombinant POx in *E. coli***.

Summarizing, the temperature of growth was the determining factor for the formation of active POx in *E. coli *(Fig. 5). At growth rates exceeding 0.25 h^-1^, volumetric activity yield decreased by at least 20% due to IB formation. Growth rates in the range of 0.02 - 0.20 h^-1 ^were favourable for the production of active POx. A sufficient amount of the inducer lactose must be provided during the whole process to guarantee permanent expression. In agreement with previous studies [22] a process temperature of 25°C turned out to be optimal for the large scale production of recombinant, active POx using this expression system in terms of both productivity [U/L·h] and process time (Fig. 4). A cultivation temperature of 25°C and induction with 0.5% (w/v) lactose from the beginning of cultivation resulted in a growth rate of μ = 0.48 h^-1 ^and a productivity rate of 32 U/L·h active POx. This result is quite different to the data achieved by Kotik et al. [22], who got a productivity rate of 140 U/L·h active POx cultivated in batches at 25°C and an induction with 0.3% (w/v) lactose. This could result from the different expression vector providing the *trc *promoter, used by Kotik et al. The *trc *promoter, which is a hybrid of the *lac *and the *trp *promoter, is known to be a weaker promoter than the strong T7 promoter, resulting in a reduced production of recombinant protein. Thus, this promoter system gives the *E. coli *folding machinery more time to successfully process recombinant proteins, instead of producing inactive IB. For the T7 promoter system instead, even at 25°C, the large amounts of POx overwhelm the folding machinery of *E. coli*, resulting in much higher IB formation. A detailed comparative study of these two promoter systems, which has previously shown this result, can be found elsewhere [34].

### Expression of POx using the pCOLD expression system

The expression vector pCOLD III drives protein expression from the promoter of the cold-shock gene *cspA*. Downstream of the *cspA *promoter, a *lac *operator is inserted so that the expression is controlled by two factors: presence of the inducer IPTG (or lactose) and by cold-shock (see manufacturer's protocol). Lactose concentrations of 5 g/L (0.5%) were found to be sufficient (data not shown). Bacteria were allowed to grow uninduced at 37°C up to certain, different levels of OD_600 _(OD_600 _= 0.5 or 2.5, respectively) before culture broths were cooled down to the final process temperatures of 25, 20 and 15°C, respectively. Addition of the inducer lactose was done simultaneously with temperature downshift. The different conditions and amounts of soluble, active POx are summarized in Table 2. In fermentation 1, POx expression was strictly growth associated and only observed during exponential growth. In total, 330 U/L of active and soluble enzyme were produced. The maximal growth rate after chilling the culture broth to 25°C was determined at 0.23 h^-1^, which is half the value compared to the pET21d^+ ^expression system under equal conditions. Glycerol depletion 26 h after inoculation resulted in an irreversible stop of POx production. Process time was kept considerably short. In fermentation 2 cells were grown to a higher optical density prior to induction, which did not result in higher yields of active POx. The amount of active POx was 190 U/L, only 40% of the yield in the first experiment. Again, expression of POx turned out to be strictly growth associated and therefore stopped upon glycerol depletion (13 h in this experiment). Apparently, the time of induction had a decisive influence on the performance of this expression system; only when exponentially growing cell cultures were induced, active POx was expressed.

**Table 2:** Growth parameters and results of batch fermentations at different temperatures using the pCOLD III/POx expression system.

fermentation	1	2	3	4	5	6
OD_600 _at induction	0.5	2.5	0.5	2.5	0.5	2.5
temp. [°C]	25	25	20	20	15	15
DCW [g/L]	4.9	5.0	4.8	4.6	4.4	4.6
Yx/s [g/g]	0.47	0.49	0.46	0.47	0.43	0.45
μ_max _[h^-1^]	0.23	0.22	0.11	0.11	0.06	0.05
vol. activity [U/L]	330	190	360	110	580	220
process time [h]	17	13	31	15	49	28
productivity [U/L·h]	19	15	12	7	12	8
space time yield [mg/L·h]	2.4	1.9	1.5	0.9	1.5	1

Protein expression was induced by providing 5 g/L (0.5% w/v) lactose at different times.

Fermentations 3 and 4 were performed at 20°C. Selecting 20°C as the final downshift temperature did not result in significantly higher expression rates of POx from pCOLD. The specific growth rate μ_max _was remarkably reduced relative to fermentations run at 25°C, which led to a doubled process time. Fermentation 4 confirmed that the point of induction dramatically affected product yield. Peak POx activity was determined with only 110 U/L, which was less than 60% compared to fermentation 2. This outcome supported the assumption that POx was only produced by actively growing biomass. Using the pCOLD expression system and growing cells to elevated densities before induction resulted in reduced process times but strongly counteracted product formation. Therefore it seems rational to initiate POx expression early in the process at low cell densities to maximize enzyme production even though this will extend process times.

**Figure 6:**
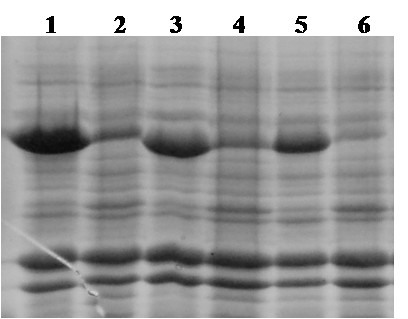
**SDS-PAGE analysis of IB formation during fermentations at different temperatures and different times of induction using the pCOLD III/POx expression system**. After lysis, cell debris containing IB was collected by centrifugation; 100 mg cell debris were washed, resuspended in Laemmli Buffer, heated at 95°C for 5 min and 10 μl aliquots were loaded per lane. Lane 1, 25°C and induction at OD_600 _0.5; Lane 2, 25°C and induction at OD_600 _2.5; Lane 3, 20°C and induction at OD_600 _0.5; Lane 4, 20°C and induction at OD_600 _2.5; Lane 5, 15°C and induction at OD_600 _0.5; Lane 6, 15°C and induction at OD_600 _2.5.

Fermentation 5 and 6 were performed at 15°C resulting in significantly higher yields of active POx. Compared to fermentation 1, in fermentation 5 the amount of active and correctly folded enzyme was increased by 75%, resulting in a final activity of 580 U/L. As shown by SDS-PAGE (Fig. 6), the amount of formed IB decreased significantly at lower temperatures. However, prolonged duration of the process poses a counterbalance to the increase in activity.

Summarizing, it can be said that performing fermentations at low temperatures is a valuable strategy to maximize enzyme production from this promoter system, although it has to be carefully estimated whether increased product formation compensates for an extended process time.

### Fed-batch fermentation using the pCOLD III/POx expression system

**Figure 7:**
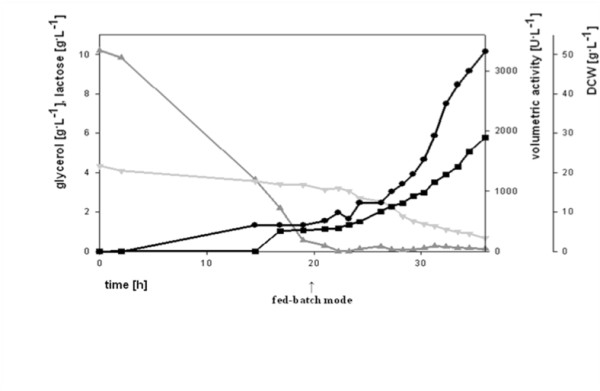
**Fed-batch fermentation at 25°C with the pCOLD III/POx expression system**. (Dark grey triangle), glycerol; (Dark grey triangle), lactose; (Black square), dry cell weight; (Black circle), volumetric activity of POx.

When the pCOLD expression system was used in batch fermentations, recombinant POx was only produced by exponentially growing cells. Therefore, achieving high cell densities by applying a fed-batch strategy seemed to be reasonable for maximizing the enzyme yield. The bioreactor was operated at a constant temperature of 25°C throughout the process and the medium was supplemented with lactose for induction from the start. We chose 25°C as process temperature for the fed-batch fermentation because experiments in batch mode had shown that at this temperature the process time was reasonable compared to the yield of active POx. Results and important parameters of the performed high cell density cultivation using the pCOLD system in a fed-batch mode are shown in Table 3 and Fig. 7. Feeding was initiated 20 h after inoculation and maintained for 16 h, resulting in a total process time of 36 h. During fed-batch mode the culture grew with a controlled specific growth rate of μ = 0.15 h^-1^, which resulted in a reasonable short process time and low IB formation. At the end of the batch phase activity reached 440 U/L, but when feeding was initiated and growth was resumed, activity levels increased exponentially with biomass. Within 16 h of feeding, POx activity rose to 3300 U/L, which was 10 times the amount of active and soluble enzyme compared to the batch experiments performed at 25°C. This result underlines the strictly growth associated production of POx. Compared to the amount of soluble, active enzyme, the overall process time was kept significantly short. The highest POx activity in batch experiments using the pCOLD system was 580 U/L after 49 h of cultivation. Here, more than 5.5 times the amount of active enzyme was produced within only 36 h of fermentation.

**Table 3:** Important parameters for the fed-batch fermentation of pCOLD III/POx at 25°C.

	Batch phase	fed-batch phase
DCW	5.7	28.9
Yx/s	0.55	0.47
μ_max _[h^-1^]	0.24	0.15
vol. activity [U/L]	440	3300
process time [h]	20	16
productivity [U/L·h]	22	206
space time yield [mg/L·h]	2.8	25.8

Protein expression was induced by addition of lactose (0.5% w/v) from the start of the cultivation.

## Conclusions

In this study we investigated the heterologous expression of the fungal enzyme POx in the prokaryotic host *E. coli *using the T7 RNA polymerase promoter-based pET21d^+^, the L-arabinose inducible pBAD and the pCOLD expression system. In preliminary experiments in baffled flasks at 25°C and optimal induction, the pBAD system turned out to be not competitive, the productivity rate of active POx was 5- and 2.3- fold lower than in the other two systems. In subsequent studies with the pET21d^+ ^and the pCOLD system using different conditions for batch fermentations, we optimized the fermentative production of POx at the bioreactor level by minimizing the formation of inactive inclusion bodies. The temperature of growth was the determining factor for the production of active POx in *E. coli*. A growth rate in the range of 0.02 - 0.20 h^-1 ^was favorable for the expression of active POx. A process temperature of 25°C turned out to be optimal for the large scale production of recombinant, active POx using both expression systems, pET21d^+ ^and pCOLD, regarding productivity [U/L·h] and process time. Finally, to increase the amount of actively expressed POx, we applied a fed-batch strategy to the pCOLD system at 25°C with a growth rate of 0.15 h^-1^. Using this system we achieved the highest productivity rate of active POx with 206 U/L·h, which was 10-fold higher than the values achieved in batch fermentations. Thus, the fed-batch strategy can be regarded superior to the simple batch mode of fermentation when it comes to produce high levels of active POx within short process times.

## Competing interests

The authors declare that they have no competing interests.

## Authors' contributions

OS and RL designed the experiments, OS and GP performed the cultivations and analyzed data, OS drafted the manuscript, DH and CKP conceived of the study, supervised research and wrote the paper. All authors read and approved the final manuscript.
